# Supplementary material for: Human papillomavirus vaccine efficacy against invasive, HPV-positive cancers: population-based follow-up of a cluster-randomised trial
Source: BMJ Open. 2021 Dec 30;11(12):e050669. doi: 10.1136/bmjopen-2021-050669 (PMC8719207; doi:10.1136/bmjopen-2021-050669)
Supplement: Author's manuscript [file bmjopen-2021-050669.draft_revisions.pdf]

BMJ Open is committed to open peer review. As part of this commitment we make the peer review history of every article we publish publicly available.

When an article is published we post the peer reviewers' comments and the authors' responses online. We also post the versions of the paper that were used during peer review. These are the versions that the peer review comments apply to.

The versions of the paper that follow are the versions that were submitted during the peer review process. They are not the versions of record or the final published versions. They should not be cited or distributed as the published version of this manuscript.

BMJ Open is an open access journal and the full, final, typeset and author-corrected version of record of the manuscript is available on our site with no access controls, subscription charges or pay-per-view fees (http://bmjopen.bmj.com).

If you have any questions on BMJ Open's open peer review process please email info.bmjopen@bmj.com

BMJ Open

Human papillomavirus vaccination protects against invasive, HPV-positive cancers

Journal:	*BMJ Open*
Manuscript ID	bmjopen-2021-050669
Article Type:	Original research
Date Submitted by the Author:	02-Mar-2021

Complete List of Authors:	Lehtinen, Matti; Karolinska Institute; Tampere University Hospital<br>Lagheden, Camilla; Karolinska Institutet, Department of Laboratory Medicine<br>Luostarinen, Tapio; Finnish Cancer Registry<br>Eriksson, Tiina<br>Apter, Dan; VL Medi<br>Bly, Anne; Tampere University Hospital<br>Gray, Penelope; Karolinska Institute<br>Harjula, Katja; Tampere University Hospital<br>Heikkilä, Kaisa; Tampere University Hospital<br>Hokkanen, Mari; Tampere University Hospital<br>Karttunen, Heidi; Tampere University Hospital<br>Kuortti, Marjo; Tampereen Yliopisto, Faculty of Social Sciences<br>Nieminen, Pekka; Helsingin Yliopisto<br>Nummela, Mervi; Tampere University Hospital<br>Paavonen, J; University of Helsinki, Dep of Gynecology and obstetrics<br>Palmroth, Johanna; University of Eastern Finland School of Medicine<br>Petäjä, Tiina; Tampereen Yliopisto, Obst&Gyn<br>Pukkala, Eero; Cancer Society of Finland<br>Soderlund-Strand, Anna; Lund University, Clinical Microbiology<br>Veivo, Ulla; Tampere University Hospital<br>Dillner, Joakim; Karolinska Institutet, Department of Medical Epidemiology and Biostatistics

Keywords:	Gynaecological oncology < ONCOLOGY, PUBLIC HEALTH, PREVENTIVE MEDICINE, SEXUAL MEDICINE

SCHOLARONE™
Manuscripts

I, the Submitting Author has the right to grant and does grant on behalf of all authors of the Work (as defined in the below author licence), an exclusive licence and/or a non-exclusive licence for contributions from authors who are: i) UK Crown employees; ii) where BMJ has agreed a CC-BY licence shall apply, and/or iii) in accordance with the terms applicable for US Federal Government officers or employees acting as part of their official duties; on a worldwide, perpetual, irrevocable, royalty-free basis to BMJ Publishing Group Ltd ("BMJ") its licensees and where the relevant Journal is co-owned by BMJ to the co-owners of the Journal, to publish the Work in this journal and any other BMJ products and to exploit all rights, as set out in our licence.

The Submitting Author accepts and understands that any supply made under these terms is made by BMJ to the Submitting Author unless you are acting as an employee on behalf of your employer or a postgraduate student of an affiliated institution which is paying any applicable article publishing charge ("APC") for Open Access articles. Where the Submitting Author wishes to make the Work available on an Open Access basis (and intends to pay the relevant APC), the terms of reuse of such Open Access shall be governed by a Creative Commons licence – details of these licences and which Creative Commons licence will apply to this Work are set out in our licence referred to above.

Other than as permitted in any relevant BMJ Author's Self Archiving Policies, I confirm this Work has not been accepted for publication elsewhere, is not being considered for publication elsewhere and does not duplicate material already published. I confirm all authors consent to publication of this Work and authorise the granting of this licence.

For peer review only

Human papillomavirus vaccination protects against invasive, HPV-positive cancers

Matti Lehtinen[1,2], Camilla Lagheden[1], Tapio Luostarinen[3], Tiina Eriksson[2], Dan Apter[4], Anne Bly[2], Penelope Gray[1], Katja Harjula[2], Kaisa Heikkilä[2], Mari Hokkanen[2], Heidi Karttunen[2], Marjo Kuortti[2], Pekka Nieminen[5], Mervi Nummela[2], Jorma Paavonen[5], Johanna Palmroth[6], Tiina Petäjä[2], Eero Pukkala[2,3], Anna Söderlund-Strand[7], Ulla Veivo[2], Joakim Dillner[1]

[1]Karolinska Institute, Stockholm, Sweden; [2]Tampere University Hospital, Tampere, Finland; [3]Finnish Cancer Registry, Helsinki, Finland; [4]VL-Medi, Helsinki, Finland; [5]University of Helsinki, Finland; [6]University of East-Finland, Kuopio, Finland; [7]Skåne University Hospital, Lund, Sweden

Funding Finnish Cancer Society, Swedish Cancer Society, Nordic Cancer Union, and GlaxoSmithKline Biologicals SA (study identifier 115006) supported the study which discloses in the publication all the data on invasive cancers collected. GlaxoSmithKline Biologicals SA was provided the opportunity to review a preliminary version of this manuscript for factual accuracy, but the authors are solely responsible for final content and interpretation.

Address for correspondence: Matti Lehtinen, Tampere University Hospital/FICAN-Mid, Tampere, Finland; E-mail: matti.lehtinen@tuni.fi

For peer review only

ABSTRACT

Background Human papillomavirus (HPV) vaccination protects against HPV, a necessary risk factor for cervical cancer. We now report results from population-based follow-up of randomized cohorts that vaccination provides HPV-type-specific protection against invasive cancer.

Methods Individually and/or cluster randomized cohorts of HPV vaccinated and non-vaccinated women were enrolled in 2002-2005. HPV-vaccine cohorts comprised originally 16-17 years-old HPV16/18 vaccinated PATRICIA (NCT00122681) and 012 trial (NCT00169494) participants (2,465), and HPV6/11/16/18 vaccinated FUTURE II (NCT00092534) participants (866). Altogether 3,341 vaccinees were followed by the Finnish Cancer Registry in the same way as 16,526 non-HPV vaccinated controls. The control cohort stemmed from 15,665 originally 18-19 years-old women enrolled in 2003 (6,499) or 2005 (9,166), and 861 placebo recipients of the FUTURE II trial. The follow-up started 6 months after the clinical trials in 2007 and 2009, and ended in 2019. It was age-aligned for the cohorts.

Findings During a follow-up time of up to 11 years we identified 17 HPV-positive invasive cancer cases (14 cervical cancers, 1 vaginal cancer, 1 vulvar cancer and 1 tongue cancer) in the non-HPV vaccinated cohorts, and no cases in the HPV-vaccinated cohorts. HPV-typing of diagnostic tumour blocks found HPV16 in 9 cervical cancer cases, HPV18, HPV33 and HPV52 each in two cases, and HPV45 in one cervical cancer case. The vaginal, vulvar and tongue cancer cases were respectively positive for HPV16, HPV52/66 and HPV213. Intention-to-treat vaccine efficacy against all HPV-positive cancers was 100% (95%CI 2,100, $p<0.05$).

Interpretation Vaccination is effective against invasive HPV-positive cancer.

For peer review only

STRENGHTS AND LIMITATIONS OF THIS STUDY

- Individually and/or age-cluster randomized cohorts of originally adolescent females received HPV vaccination, control vaccine vaccination or no vaccination in 2002-2005.
- All study participants consented to unique personal identifier facilitated long-term follow-up by the population-based Finnish Cancer Registry.
- All study participants had a possibility to attend cervical screening at the age of 25 and 30 years.
- All diagnostic biopsies with cervical or other HPV-associated cancers were retrieved for HPV DNA typing.
- Our HPV-vaccinated cohorts stem from the two largest clinical phase III trials, with 10% and 25% Finnish involvement and maximum, age-aligned follow-up time of up to 15 years, but the sample sizes are limited.

For peer review only

INTRODUCTION

Human papillomavirus (HPV) vaccines are effective against cervical intraepithelial neoplasia grade 3 (CIN3) 8 to 14 years post vaccination.[1-3] The risk of invasive cervical cancer overall is lower among HPV-vaccinated women as compared to non-HPV vaccinated women.[4] From population-based cancer registry follow-up of randomized cohorts we have previously reported a significant vaccine efficacy (VE) against all invasive HPV-associated cancers.[5] We have now extended the follow-up time to 11 years and performed HPV-typing of diagnostic tumour blocks. Our objective is to report the first HPV type-specific vaccine efficacy estimates against invasive cancers in a randomized setting.

For peer review only

METHODS

Enrolment, intervention and follow-up All the 22,412 Finnish women born in annual quartal (Q)4/1984-Q1/1987 were invited to participate the FUTURE II (NCT00092534)[6] phase III HPV6/11/16/18 vaccine trial at age 16 to 17 years in seven towns in Q4/2002-Q1/2003. Corresponding 24,064 Finnish women born Q2/1986-Q1/1988 were invited to participate the PATRICIA (NCT00122681)[7] and HPV-012 (NCT00337818)[1] vaccine trials also at the age 16 to 17 years in eighteen Finnish towns in Q2/2004-Q1/2005 (figure 1).

There were 3,341 16-17 years-old HPV vaccinated trial participants (by trial: 866, 2,475 (2,409 + 66).[1,6,7] As controls we had altogether 16,526 16-19 years-old non-HPV-vaccinated females enrolled, respectively in June 2003/2005 (15,655 18-19 year-olds)[6,7] and during Q4/2002-Q1/2003 (861 16-17 years-old placebo recipients of the FUTURE II trial)[7] (figure 1).

In June 2003 and June 2005 all (30,947 and 58,996) non-HPV-vaccinated, 18 and 18-19 year-old Finnish females from the entire, immediately adjacent birth cohorts, born respectively in Q3/1984-Q2/1985 and Q3/1985-Q2/1987, not eligible to the above-mentioned clinical trials, were invited to participate in Finnish Cancer Registry (FCR) –based follow-up (figure 1).[6,7] Participation in an HPV-vaccination trial was an exclusion criterion.

All the clinical trial participants and the non-HPV-vaccinated controls consented to the FCR-based follow-up for invasive, histopathologically confirmed cancer outcome.[1,6-10] The follow-up was based on unique Finnish personal identifiers available at our study database following the informed consent, and generally used at the FCR. It was age-aligned[8] to ensure that all the originally 16-17 years-old HPV-vaccinated women and originally 16-19 years-old non-HPV vaccinated women were of similar age during comparable follow-up time-period of 11 years between 2007 and 2019.

For peer review only

All participants of the FCR-based follow-up were invited to cervical screening at the ages of 25, 30 and 35 years. For those with abnormal cytology during the trials, there was referral to colposcopy with biopsy within 6 months[1] and the FCR follow-up thus started 6 months after the trials' end. All the cohorts responded at the age of 22 years to a life-style questionnaire, with special emphasis on sexual health and previous HPV-vaccination. Death and emigration were elimination criteria. For the non-HPV-vaccinated any identified HPV- vaccination was an elimination criteria. All the clinical HPV-vaccination trials, and the long-term follow-up of the trial cohorts and the non-HPV-vaccinated cohort were approved by the Finnish National Ethical Review Board (ERB, TUKIJA 1150/2002, 1153/2003, 1174/2004).

Patient and Public Involvement Patients were not involved in this preventive intervention study that involved originally adolescent HPV vaccinated or unvaccinated young women. To guarantee public involvement the entire target population was duly informed by letters using the up to date address service of the Finnish Population Register. The trial invitations were mailed to parents or guardians of all adolescents girls of the above-mentioned birth cohorts and living in the same house-hold. This was done even if the girl's own informed consent was sufficient for participation according to the Finnish law. Public involvement in the enrolment was further assured by information lectures given by the study nurses at parental evenings of the secondary high-schools and technical schools in the seven and eighteen trial communities.

Laboratory analyses The diagnostic histopathological blocks were requested from the pathology laboratories that had notified the FCR about the incident cancer cases according to a specific permission from the Finnish National Supervisory Authority for Welfare and Health (Valvira) without informing the patients. Before HPV DNA-typing the presence of neoplastic tissue was re-reviewed by two experienced pathologists. Blocks were sectioned according to a contamination-

For peer review only

proof manner. Extraction, amplification, and typing of the intralesional HPV DNA was performed as previously described.[11,12] HPV types that remained unknown were identified by sequencing.

Statistical analyses The enrolled HPV vaccinated and unvaccinated adolescents provided 80% power for the identification of vaccine efficacy (VE) against cervical cancer.[6,7] The VE was calculated on the intention-to-treat principle including all individuals (regardless of baseline HPV status) who had received at least one vaccine dose in the HPV-vaccine arm. Statistical software SAS 9.4 software (SAS Institute, Cary, NC) was used. The 95% confidence intervals (95% CI) were based on exact binomial distribution of number of vaccinated cases conditional on total number of cases.[13]

For peer review only

RESULTS

The age-aligned Finnish Cancer Registry follow-up for incident invasive cancers lasted by cohort for up to 11 years between November 2007 and Dec 2019. According to Finnish Population Census Register altogether 10 FUTURE II, 19 PATRICIA and 73 non-HPV vaccinated participants died during the follow-up (figure 1). It resulted in respectively 33,792 and 174,340 follow-up years for the HPV-vaccine and non-HPV vaccine cohorts.

No HPV-associated cancer cases were found in the 3,341 HPV-vaccinated females through the 33,792 women years of the follow up (table 1). The follow-up identified in the 16,526 non-HPV-vaccinated women 15 cases with invasive cervical cancer (ICC) in the 16,526 non-HPV vaccinated females during 174,320 women years of follow-up. Four of the ICC cases were adenocarcinomas and 11 were squamous cell carcinomas (SCC). One cervical SCC case, diagnosed late in 2019, was outside the age-aligned time-window of 11 years that guaranteed equal follow-up for the different cohorts. This case was not included in further analyses. The incidence of the 14 eligible ICC cases in the non-HPV-vaccinated women was 8.0 per 100,000 women years (table 1).

The single, invasive vaginal, vulvar and tongue cancer cases were all squamous cell carcinomas. Overall, in the non-HPV-vaccinated women the incidence of the HPV-associated cancers was 9.8 per 100,000 women years. Incidence rates of breast cancer, thyroid cancer and melanoma were essentially similar in all cohorts with highly overlapping 95% confidence intervals (table 1).

HPV DNA was found in all the identified and eligible 17 invasive HPV-associated cancer cases (table 2). HPV16 was present in 9 invasive cervical cancer cases. HPV16 was present in one female with vaginal cancer, who also had HPV16 positive cervical cancer. HPV18, HPV33 and HPV52 were each present in two ICC cases, and HPV45 in one ICC case. HPV66 (a vulvar cancer also positive for HPV52) and HPV213 (a tongue cancer) were present in one case each (table 2). With

For peer review only

no identified HPV-associated cancer cases in the HPV-vaccinated females, we found a vaccine efficacy of 100% (95% confidence interval 2,100; $p<0.05$) against all HPV DNA-positive invasive cancers.

For peer review only

DISCUSSION

We report the first randomized trial –derived evidence on the significant, 100% HPV-vaccine efficacy against HPV DNA-positive invasive cancers. We followed both the bivalent and the quadrivalent HPV-vaccine recipients for 11 years after the clinical trials had ended (up to 17 years post vaccination), and similarly-aged non-HPV vaccinated females, respectively over approximately 35,000 and 175,000 follow-up years by the population-based, quality-controlled Finnish Cancer Registry.[9,10] Vaccine efficacy estimates against invasive HPV16/18 positive cancers, and against invasive cancer cases positive for the bivalent vaccine cross-protected HPV types 33/45/52[14-16] were all 100%, albeit with very wide confidence intervals.

The significant 100% VE against all HPV-positive cancers is reassuring for both the bivalent and quadrivalent HPV-vaccines, three shots of which were given to, respectively 26% and 74% of the vaccinees in 2002 and 2004. A recent Swedish study[4] which was also based on accurate, high quality cancer registry follow-up[13] suggested that up to 12% of invasive cervical cancer cases may not be prevented by the quadrivalent vaccine. We have recently shown that long-term cross-neutralizing antibody responses induced by these two vaccines differ significantly.[16] Thus, continuation of the cancer registry-based long-term follow-up of the population-based bivalent and the quadrivalent HPV-vaccine cohorts versus non-HPV vaccinated cohorts[6,7] is warranted to understand if there were a possible difference their efficacy.

Population-based, age-aligned follow-up of trial cohorts and accurate, histologically confirmed, invasive cancer case definitions of the quality-controlled Finnish Cancer Registry[6-8,12,13,17] were pivotal strengths of this study. We could ensure identical cervical screening history of HPV vaccinated and non-HPV vaccinated cohorts for the 1983-1988 birth cohorts that were included in this study. Opportunistic HPV vaccination among the unvaccinated controls was negligible.[18,19]

For peer review only

During 2007-2009 entire 1992-1995 birth cohorts of 33 Finnish towns were enrolled in our population-based community-randomized HPV-vaccination trial.[20,21] HPV16/18 vaccine recipients of that trial have since 2014 participated in a re-randomized screening trial at ages 22, 25 and 28 years[22], while their non-HPV vaccinated counterparts have not had this opportunity. These sizeable cohorts, >40,000 individuals in total, were not included in this study due to their gradually more and more different screening histories.

Two independent pathologists ensured that neoplastic tissue was present in the sections of the initial diagnostic biopsy blocks that were used for HPV testing. In three ICC cases, CIN3 was left in the diagnostic block that was used for HPV DNA typing but the sectioned material was deemed to adequately represent oncogenic HPV types involved. The blocks were found to be positive for HPV16, HPV33 and HPV16/52 DNA. Conceivably, prior sections had removed an invasive cervical cancer before the new sections were made for the HPV DNA typing.

Evidence on protection against the most stringent, invasive HPV-associated cervical cancer end-point is now emerging more rapidly[3] than the evidence on the efficacy of HBV vaccination against hepatocellular cancer. [23] HPV-cancers in young adults may be more rapidly developing. However, establishing clinical trial cohorts and concomitant control cohorts, and their follow-up up to 17 years ago with identical possibilities for organized cervical screening at ages 25, 30 and 35 years[1,17] has been pivotal. Recruiting the different cohorts in a population-based fashion during 2002-2005 was also important prerequisite for the country-wide cancer registry –based follow-up.[6-8,17]

The excellent vaccine efficacy against HPV-positive cancers, now documented from a randomized study setting, is an important evidence of the long-term impact of HPV vaccination, and paves the way for the WHO's initiative on the elimination of cervical cancer.[24]

For peer review only

Acknowledgements The review of diagnostic histopathological blocks for HPV DNA analysis by drs. Mensur Dzabic and Ralf Butzow is gratefully acknowledged.

Funding GSK Biologicals SA (HPV-027) and Finnish Cancer Foundation (MS790) supported the study financially,

Conflicts of interes We declare that ML, DA, JD and JP have received grants from Merck & Co. Inc. and/or from the GSK group of companies through their respective employers. GSK Biologicals SA was provided the opportunity to review this manuscript but the authors are solely responsible for final content and interpretation.

Contributorship The corresponding author attests that all listed authors meet authorship criteria and that no others meeting the criteria have been omitted. Matti Lehtinen, Tapio Luostarinen, Dan Apter, Joakim Dillner, Tiina Eriksson, Jorma Paavonen, and Eero Pukkala were involved in the conception and/or the design of the study. Matti Lehtinen, Dan Apter, Anne Bly, Tiina Eriksson, Katja Harjula, Kaisa Heikkilä, Mari Hokkanen, Heidi Karttunen, Marjo Kuortti, Tapio Luostarinen, Pekka Nieminen, Mervi Nummela, Johanna Palmroth, Tiina Petäjä, Anna Söderlund-Strand, and Ulla Veivo participated in the collection and generation of the study data. Tiina Eriksson, Penelope Gray, Camilla Lagheden and Tapio Luostarinen performed the analyses. Matti Lehtinen, Dan Apter, Tapio Luostarinen, Jorma Paavonen, and Eero Pukkala were involved in the analyses and/or interpretation of the data. All authors contributed to development of this manuscript, had full access to the data and gave final approval before submission.

Transparency declaration The leading author affirms that the manuscript is an honest, accurate, and transparent account of the study being reported; that no important aspects of the study have been omitted; and that any discrepancies from the study as planned and registered have been explained.

For peer review only

REFERENCES

1. Lehtinen M, Lagheden C, Luostarinen T, et al. Ten-year follow-up of human papillomavirus vaccine efficacy against the most stringent cervical neoplasia end-point – registry-based follow-up of three cohorts from randomized trials. *BMJ Open* 2017;7:e015867.

2. Kjaer SK, Nygard M, Sundstrom K, et al. Final analysis of a 14-year long-term follow-up study of the effectiveness and immunogenicity of the quadrivalent human papilloma virus vaccine in women from four Nordic countries. *EClinMed* 2020; 23:100401.

3. Kjaer SK, Nygard M, Sundstrom K, et al. Long-term effectiveness of the ninevalent human papilloma virus vaccine in Scandinavian women. *Human Vac. Immunother.* 2020; doi:10.1082/216455.2020.1839292

4. Lei J, Ploner A, Elfström KM, et al. HPV vaccination and the risk of invasive cervical cancer. *N Engl J Med* 2020;383:1340-8.

5. Luostarinen T, Apter D, Diller J, et al. Vaccination protects against invasive HPV-associated cancer. *Int J Cancer* 2018;142:2186-7.

6. Lehtinen M, Idänpään-Heikkilä I, Lunnas T, et al. Population-based enrolment of adolescents in a long-term follow-up trial of HPV vaccine efficacy. *Int J STD AIDS* 2006;17:237-46.

7. Lehtinen M, Apter D, Dubin G, et al. Enrolment of 22,000 adolescent women to cancer registry follow-up for long-term human papillomavirus vaccine efficacy. *Int J STD AIDS* 2006;17:517-21.

8. Rana M, Huhtala H, Apter D, et al. Understanding long-term protection of HPV vaccination against cervical carcinoma: Cancer registry-based follow-up. *Int J Cancer* 2012;132:2833-8.

9. Korhonen P, Malila N, Pukkala E, et al. The Finnish Cancer Registry as follow-up source of a large trial cohort-accuracy and delay. *Acta Oncol* 2002;41:381-8.

10. Pukkala E , Engholm G, Højsgaard Schmidt LK, et al. Nordic Cancer Registries - an overview of their procedures and data comparability. *Acta Oncol* 2018;57;440-55.

For peer review only

11. Lagheden C, Eklund C, Kleppe SN, et al. Validation of a standardized extraction method for formalin-fixed paraffin-embedded tissue samples. *J Clin Virol* 2016;80:36-9.

12. Lagheden C, Eklund C, Lamin H, et al. Nationwide comprehensive human papillomavirus (HPV) genotyping of invasive cervical cancer. Br J Cancer 2018; **118**: 1377-81.

13. Ewell M. Comparing methods for calculating confidence intervals for vaccine efficacy. *Stat Med* 1996;15:2379-92.

14. Wheeler CM, Castellsague X, Garland S, et al. Efficacy of the HPV-16/18 AS04-adjuvanted vaccine against cervical infection and precancer caused by non-vaccine oncogenic HPV types (PATRICIA trial). *Lancet Oncol* 2012;13:100-10.

15. Bogaards JA, vad der Weele P, Woestenberg PJ, et al. Bivalent HPV vaccine effectiveness correlates with phylogenetic distance from HPV vaccine types 16 and 18. *J Infect Dis* 2019;220: 1141-6.

16. Mariz F, Bender N, Eriksson T, et al. Sustainability of bi- and quadrivalent HPV vaccine-induced neutralizing antibodies. *Lancet Infect Dis* 2020; in press.

17. Lehtinen M, Herrero R, Mayaud P, et al. Studies to assess long-term efficacy and effectiveness of HPV vaccination in developed and developing countries. *Vaccine* 2006;24S:233-41.

18. Woodhall SC, Eriksson T, Nykanen A-M, et al. Impact of HPV vaccination on quality of life. *Eur J Contracept Reproduct Health Care* 2011;16:3-8.

19. Eriksson T, Torvinen, Lehtinen MM, et al. Impact of HPV16/18 vaccination on quality of life. *Eur J Contracept Reproduct Health Care* 2013;18:364-71.

20. Lehtinen M, Baussano I, Apter D, et al. Characteristics of a cluster-randomized, phase IV human papillomavirus vaccination effectiveness trial. *Vaccine* 2015;33:1284-90.

21. Vänskä S, Luostarinen T, Baussano I, et al. Vaccination with moderate coverage eradicates oncogenic papillomaviruses if a gender-neutral strategy is applied. *J Infect Dis* 2020;222:948-56.

For peer review only

22. Louvanto K, Eriksson M, Elfströn M, et al. Effectiveness of screening in human papillomavirus vaccinated women. *Int J Cancer* 2020;147:440-7.

23. McMahon BJ, Bulkow LR, Singleton RJ, et al. Elimination of hepatocellular carcinoma and acute hepatitis B in children 25 years after a hepatitis B newborn and catch-up immunization program. *Hepatology* 2011;54:801-7.

24. Basu P, Hutubessy R, Broutet N. Cervical cancer: WHO called for elimination, not eradication *BMJ* 2019;366doi:https://doi.org/10.1136/bmj.l5668.

For peer review only

Figure 1. Study flow chart defines the non-overlapping birth cohorts enrolled to long-term follow-up of human papillomavirus vaccination trials involving the quadrivalent HPV6/11/16/18 vaccine (FUTURE II) or the bivalent HPV16/18 vaccine (PATRICIA and HPV-012), or two non-HPV vaccinated control cohorts by population-based invitations via Finnish Population Register in 2002-05. Q=annual quartal.

For peer review only

Table 1. Numbers (n) and incidence rates (/100,000 person years) of human papillomavirus (HPV) associated invasive cancers and other common cancers in cluster-randomized cohorts of altogether 3,341 16-17 year-old female HPV16/18 or HPV6/11/16/18 vaccine recipients and 16,526 non-HPV vaccinated, originally 16-19 year-old females followed up§ between 2007 to 2019.

	HPV vaccinated women (33,792 person years)		Non-HPV vaccinated women (174,340 person years)	
End-point	n	Rate (95%CI)	n	Rate (95% CI)
Cervical cancer	0	-	14	8.0 (4.8, 13.6)
All HPV-positive cancers*	0	-	17	9.8 (6.1, 15.7)
Breast cancer	3	8.9 (2.9, 28)	27	15.5 (10.6, 23)
Thyroid cancer	2	5.9 (1.5, 24)	16	9.2 (5.6, 15.0)
Melanoma	8	23.8 (11.8, 47)	22	12.6 (8.3, 19.2)

§For corresponding age-aligned sub-cohorts, up to 11-years of passive follow-up was by the population-based Finnish Cancer Registry.
*14 cervical cancers, one vaginal cancer, one vulvar cancer, one tongue cancer

For peer review only

Table 2. Numbers (n) and incidence rates (/100,000 person years) of human papillomavirus (HPV) positive invasive cancers by HPV type in cluster-randomized cohorts of altogether 3,341 16-17 year-old female HPV16/18 or HPV6/11/16/18 vaccine recipients and 16,526 non-HPV vaccinated, originally 16-19 year-old females followed up§ during 2007 to 2019.

	HPV vaccinated women (33,792 person years)		Non-HPV vaccinated women (174,340 person years)	
End-point	n	Rate (95%CI)	n	Rate (95% CI)
Cervical cancer				
HPV16	0	-	9	5.2 (2.7, 9.9)
HPV18	0	-	2	1.1 (0.3, 4.6)
HPV16/18	0	-	11	6.3 (3.5, 11.4)
HPV33	0	-	2	1.1 (0.3, 4.6)
HPV45	0	-	1	0.6 (0.1, 4.1)
HPV52*	0	-	1*	0.6 (0.1, 4.1)
Any HPV	0	-	14	8.0 (4.8, 13.6)
Vaginal cancer				
HPV16	0	-	1	0.6 (0.1, 4.1)
Vulvar cancer				
HPV52#	0	-	1	0.6 (0.1, 4.1)
Tongue cancer				
HPV213	0	-	1	0.6 (0.1, 4.1)

§For corresponding age-aligned sub-cohorts, up to 11-years of passive follow-up was by the population-based Finnish Cancer Registry.
*Positive for both HPV16 and HPV52, #Positive for both HPV52 and HPV66

For peer review only

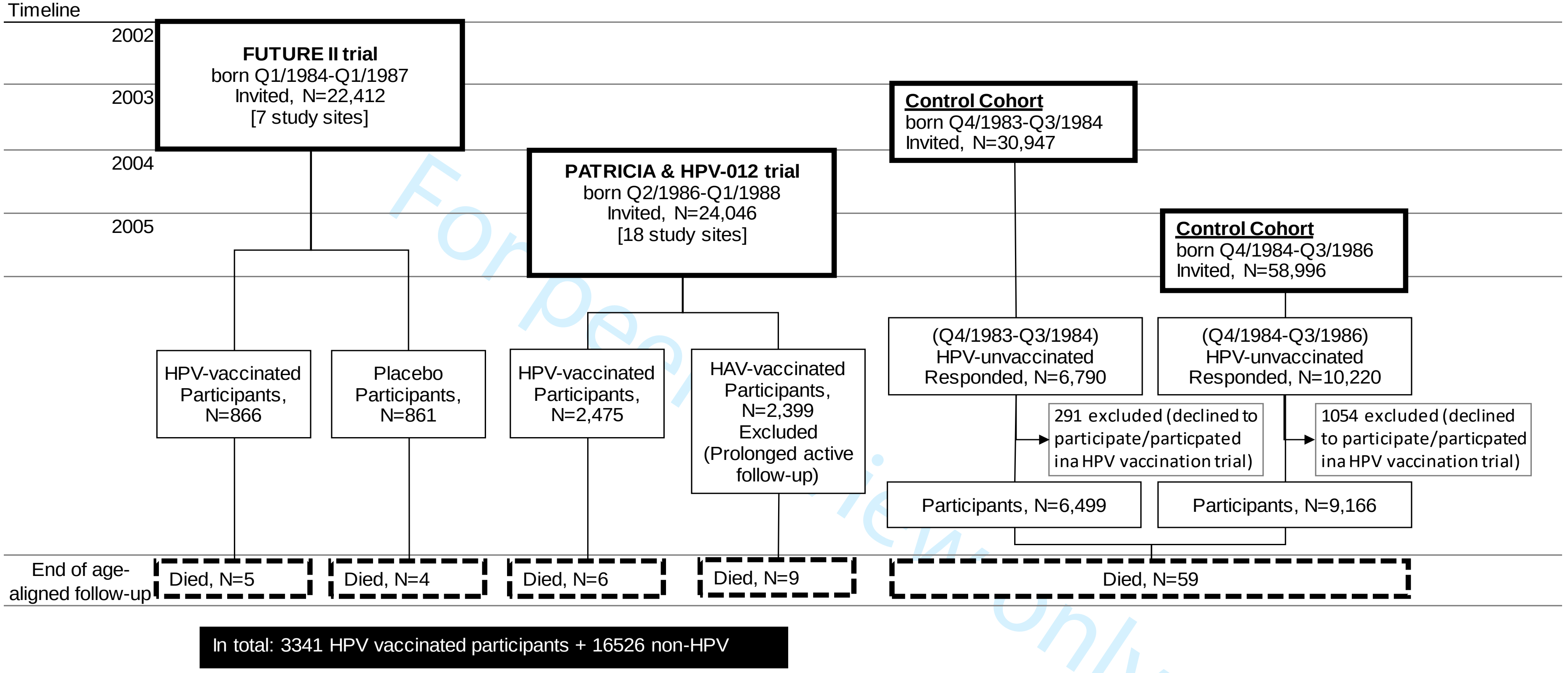
Timeline
2002
2003
2004
2005
FUTURE II trial
born Q1/1984-Q1/1987
Invited, N=22,412
[7 study sites]
PATRICIA & HPV-012 trial
born Q2/1986-Q1/1988
Invited, N=24,046
[18 study sites]
Control Cohort
born Q4/1983-Q3/1984
Invited, N=30,947
Control Cohort
born Q4/1984-Q3/1986
Invited, N=58,996
HPV-vaccinated Participants, N=866
Placebo Participants, N=861
HPV-vaccinated Participants, N=2,475
HAV-vaccinated Participants, N=2,399 Excluded (Prolonged active follow-up)
(Q4/1983-Q3/1984) HPV-unvaccinated Responded, N=6,790
(Q4/1984-Q3/1986) HPV-unvaccinated Responded, N=10,220
291 excluded (declined to participate/particpated ina HPV vaccination trial)
1054 excluded (declined to participate/particpated ina HPV vaccination trial)
Participants, N=6,499
Participants, N=9,166
End of age-aligned follow-up
Died, N=5
Died, N=4
Died, N=6
Died, N=9
Died, N=59
In total: 3341 HPV vaccinated participants + 16526 non-HPV

STROBE Statement—Checklist of items that should be included in reports of ***cohort studies***

	Item No	Recommendation	Page No
**Title and abstract**	1	(*a*) Indicate the study's design with a commonly used term in the title or the abstract	2
		(*b*) Provide in the abstract an informative and balanced summary of what was done and what was found	2
**Introduction**			
Background/rationale	2	Explain the scientific background and rationale for the investigation being reported	3
Objectives	3	State specific objectives, including any prespecified hypotheses	3
**Methods**			
Study design	4	Present key elements of study design early in the paper	3
Setting	5	Describe the setting, locations, and relevant dates, including periods of recruitment, exposure, follow-up, and data collection	4,5
Participants	6	(*a*) Give the eligibility criteria, and the sources and methods of selection of participants. Describe methods of follow-up	4,5
		(*b*)For matched studies, give matching criteria and number of exposed and unexposed	-
Variables	7	Clearly define all outcomes, exposures, predictors, potential confounders, and effect modifiers. Give diagnostic criteria, if applicable	4
Data sources/ measurement	8*	For each variable of interest, give sources of data and details of methods of assessment (measurement). Describe comparability of assessment methods if there is more than one group	4,5
Bias	9	Describe any efforts to address potential sources of bias	8, 9
Study size	10	Explain how the study size was arrived at	5
Quantitative variables	11	Explain how quantitative variables were handled in the analyses. If applicable, describe which groupings were chosen and why	
Statistical methods	12	(*a*) Describe all statistical methods, including those used to control for confounding	5
		(*b*) Describe any methods used to examine subgroups and interactions	
		(*c*) Explain how missing data were addressed	
		(*d*) If applicable, explain how loss to follow-up was addressed	
		(*e*) Describe any sensitivity analyses	
**Results**			
Participants	13*	(a) Report numbers of individuals at each stage of study—eg numbers potentially eligible, examined for eligibility, confirmed eligible, included in the study, completing follow-up, and analysed	6
		(b) Give reasons for non-participation at each stage	6
		(c) Consider use of a flow diagram	6
Descriptive data	14*	(a) Give characteristics of study participants (eg demographic, clinical, social) and information on exposures and potential confounders	6
		(b) Indicate number of participants with missing data for each variable of interest	-
		(c) Summarise follow-up time (eg, average and total amount)	6
Outcome data	15*	Report numbers of outcome events or summary measures over time	6

For peer review only - http://bmjopen.bmj.com/site/about/guidelines.xhtml

Main results	16	(*a*) Give unadjusted estimates and, if applicable, confounder-adjusted estimates and their precision (eg, 95% confidence interval). Make clear which confounders were adjusted for and why they were included<br>(*b*) Report category boundaries when continuous variables were categorized<br>(*c*) If relevant, consider translating estimates of relative risk into absolute risk for a meaningful time period	6,7
Other analyses	17	Report other analyses done—eg analyses of subgroups and interactions, and sensitivity analyses	-
**Discussion**			
Key results	18	Summarise key results with reference to study objectives	8
Limitations	19	Discuss limitations of the study, taking into account sources of potential bias or imprecision. Discuss both direction and magnitude of any potential bias	8, 9
Interpretation	20	Give a cautious overall interpretation of results considering objectives, limitations, multiplicity of analyses, results from similar studies, and other relevant evidence	2, 9
Generalisability	21	Discuss the generalisability (external validity) of the study results	9
**Other information**			
Funding	22	Give the source of funding and the role of the funders for the present study and, if applicable, for the original study on which the present article is based	1

*Give information separately for exposed and unexposed groups.

Note: An Explanation and Elaboration article discusses each checklist item and gives methodological background and published examples of transparent reporting. The STROBE checklist is best used in conjunction with this article (freely available on the Web sites of PLoS Medicine at http://www.plosmedicine.org/, Annals of Internal Medicine at http://www.annals.org/, and Epidemiology at http://www.epidem.com/). Information on the STROBE Initiative is available at http://www.strobe-statement.org.

For peer review only

BMJ Open

Human papillomavirus vaccine efficacy against invasive, HPV-positive cancers – Population-based follow-up of a cluster-randomized trial

Journal:	*BMJ Open*
Manuscript ID	bmjopen-2021-050669.R1
Article Type:	Original research
Date Submitted by the Author:	30-Sep-2021
Complete List of Authors:	Lehtinen, Matti; Karolinska Institute; Tampere University Hospital<br>Lehtinen, Matti; Karolinska Institute Department of Laboratory Medicine,<br>Lagheden, Camilla; Karolinska Institutet, Department of Laboratory Medicine<br>Luostarinen, Tapio; Finnish Cancer Registry<br>Eriksson, Tiina<br>Apter, Dan; VL Medi<br>Bly, Anne; Tampere University Hospital<br>Gray, Penelope; Karolinska Institute<br>Harjula, Katja; Tampere University Hospital<br>Heikkilä, Kaisa; Tampere University Hospital<br>Hokkanen, Mari; Tampere University Hospital<br>Karttunen, Heidi; Tampere University Hospital<br>Kuortti, Marjo; Tampereen Yliopisto, Faculty of Social Sciences<br>Nieminen, Pekka; Helsingin Yliopisto<br>Nummela, Mervi; Tampere University Hospital<br>Paavonen, J; University of Helsinki, Dep of Gynecology and obstetrics<br>Palmroth, Johanna; University of Eastern Finland School of Medicine<br>Petäjä, Tiina; Tampereen Yliopisto, Obst&Gyn<br>Pukkala, Eero; Cancer Society of Finland<br>Soderlund-Strand, Anna; Lund University, Clinical Microbiology<br>Veivo, Ulla; Tampere University Hospital<br>Dillner, Joakim; Karolinska Institutet, Department of Medical Epidemiology and Biostatistics
**Primary Subject Heading**:	Epidemiology
Secondary Subject Heading:	Infectious diseases, Oncology, Obstetrics and gynaecology
Keywords:	Gynaecological oncology < ONCOLOGY, PUBLIC HEALTH, PREVENTIVE MEDICINE, SEXUAL MEDICINE, EPIDEMIOLOGY

SCHOLARONE™ Manuscripts

For peer review only - http://bmjopen.bmj.com/site/about/guidelines.xhtml

I, the Submitting Author has the right to grant and does grant on behalf of all authors of the Work (as defined in the below author licence), an exclusive licence and/or a non-exclusive licence for contributions from authors who are: i) UK Crown employees; ii) where BMJ has agreed a CC-BY licence shall apply, and/or iii) in accordance with the terms applicable for US Federal Government officers or employees acting as part of their official duties; on a worldwide, perpetual, irrevocable, royalty-free basis to BMJ Publishing Group Ltd ("BMJ") its licensees and where the relevant Journal is co-owned by BMJ to the co-owners of the Journal, to publish the Work in this journal and any other BMJ products and to exploit all rights, as set out in our licence.

The Submitting Author accepts and understands that any supply made under these terms is made by BMJ to the Submitting Author unless you are acting as an employee on behalf of your employer or a postgraduate student of an affiliated institution which is paying any applicable article publishing charge ("APC") for Open Access articles. Where the Submitting Author wishes to make the Work available on an Open Access basis (and intends to pay the relevant APC), the terms of reuse of such Open Access shall be governed by a Creative Commons licence – details of these licences and which Creative Commons licence will apply to this Work are set out in our licence referred to above.

Other than as permitted in any relevant BMJ Author's Self Archiving Policies, I confirm this Work has not been accepted for publication elsewhere, is not being considered for publication elsewhere and does not duplicate material already published. I confirm all authors consent to publication of this Work and authorise the granting of this licence.

For peer review only

Human papillomavirus vaccine efficacy against invasive, HPV-positive cancers – Population-based follow-up of a cluster-randomized trial

Matti Lehtinen[1,2], Camilla Lagheden[1], Tapio Luostarinen[3], Tiina Eriksson[2], Dan Apter[4], Anne Bly[2], Penelope Gray[1], Katja Harjula[2], Kaisa Heikkilä[2], Mari Hokkanen[2], Heidi Karttunen[2], Marjo Kuortti[2], Pekka Nieminen[5], Mervi Nummela[2], Jorma Paavonen[5], Johanna Palmroth[6], Tiina Petäjä[2], Eero Pukkala[2,3], Anna Söderlund-Strand[7], Ulla Veivo[2], Joakim Dillner[1]

[1]Karolinska Institute, Stockholm, Sweden; [2]Tampere University Hospital, Tampere, Finland; [3]Finnish Cancer Registry, Helsinki, Finland; [4]VL-Medi, Helsinki, Finland; [5]University of Helsinki, Finland; [6]University of East-Finland, Kuopio, Finland; [7]Skåne University Hospital, Lund, Sweden

Funding Finnish Cancer Society, Swedish Cancer Society, Nordic Cancer Union, and GlaxoSmithKline Biologicals SA (study identifier 115006) supported the study which discloses in the publication all the data on invasive cancers collected. GlaxoSmithKline Biologicals SA was provided the opportunity to review a preliminary version of this manuscript for factual accuracy, but the authors are solely responsible for final content and interpretation.

Corresponding author: Matti Lehtinen, Tampere University Hospital/FICAN-Mid, Tampere 33520, Finland; E-mail: inkeri.lehtinen@gmail.com

For peer review only

ABSTRACT

Background Human papillomavirus (HPV) vaccination protects against HPV, a necessary risk factor for cervical cancer. We now report results from population-based follow-up of randomized cohorts that vaccination provides HPV-type-specific protection against invasive cancer.

Methods Individually and/or cluster randomized cohorts of HPV vaccinated and non-vaccinated women were enrolled in 2002-2005. HPV-vaccine cohorts comprised originally 16-17 years-old HPV16/18 vaccinated PATRICIA (NCT00122681) and 012 trial (NCT00169494) participants (2,465), and HPV6/11/16/18 vaccinated FUTURE II (NCT00092534) participants (866). Altogether 3,341 vaccinees were followed by the Finnish Cancer Registry in the same way as 16,526 non-HPV vaccinated controls. The control cohort stemmed from 15,665 originally 18-19 years-old women enrolled in 2003 (6,499) or 2005 (9,166), and 861 placebo recipients of the FUTURE II trial. The follow-up started 6 months after the clinical trials in 2007 and 2009, and ended in 2019. It was age-aligned for the cohorts.

Findings During a follow-up time of up to 11 years we identified 17 HPV-positive invasive cancer cases (14 cervical cancers, 1 vaginal cancer, 1 vulvar cancer and 1 tongue cancer) in the non-HPV vaccinated cohorts, and no cases in the HPV-vaccinated cohorts. HPV-typing of diagnostic tumour blocks found HPV16 in 9 cervical cancer cases, HPV18, HPV33 and HPV52 each in two cases, and HPV45 in one cervical cancer case. The vaginal, vulvar and tongue cancer cases were respectively positive for HPV16, HPV52/66 and HPV213. Intention-to-treat vaccine efficacy against all HPV-positive cancers was 100% (95%CI 2,100, $p<0.05$).

Interpretation Vaccination is effective against invasive HPV-positive cancer.

For peer review only - http://bmjopen.bmj.com/site/about/guidelines.xhtml

STRENGHTS AND LIMITATIONS OF THIS STUDY

- Individually and/or age-cluster randomized cohorts of originally adolescent females received HPV vaccination, control vaccine vaccination or no vaccination in 2002-2005.

- All study participants consented to unique personal identifier facilitated long-term follow-up by the population-based Finnish Cancer Registry.

- All study participants had a possibility to attend cervical screening at the age of 25 and 30 years.

- All diagnostic biopsies with cervical or other HPV-associated cancers were retrieved for HPV DNA typing.

- Our HPV-vaccinated cohorts stem from the two largest clinical phase III trials, with 10% and 25% Finnish involvement and maximum, age-aligned follow-up time of up to 15 years, but the sample sizes are limited.

-Opportunistic, post-licensure HPV-vaccination was negligible, probably had no effect on cervical precancer incidence but could not be totally ruled out.

For peer review only

INTRODUCTION

Human papillomavirus (HPV) vaccines are effective against cervical intraepithelial neoplasia grade 3 (CIN3) 8 to 14 years post vaccination.[1-3] The risk of invasive cervical cancer overall is lower among HPV-vaccinated women as compared to non-HPV vaccinated women.[4] From population-based cancer registry follow-up of randomized cohorts we have previously reported a significant vaccine efficacy (VE) against all invasive HPV-associated cancers.[5] We have now extended the follow-up time to 11 years and performed HPV-typing of diagnostic tumour blocks. Our objective is to report the first HPV type-specific vaccine efficacy estimates against invasive cancers in a randomized setting.

For peer review only

METHODS

Enrolment, intervention and follow-up All the 22,412 Finnish women born in annual quartal (Q)4/1984-Q1/1987 were invited to participate: 1. the FUTURE II (NCT00092534)[6] phase III HPV6/11/16/18 vaccine trial at age 16 to 17 years in seven towns in Q4/2002-Q1/2003. 24,064 women born Q2/1986-Q1/1988 were invited to participate: 2. the PATRICIA (NCT00122681)[7] phase III trial, and 3. the HPV-012 (NCT00337818)[1] phase II trial also at the age 16 to 17 years in eighteen Finnish towns in Q2/2004-Q1/2005 (figure 1). There were 3,341 16-17 years-old HPV vaccinated trial participants (by trial: 1. 866, 2. 2,409 and 3. 66) (figure 1).[1,6,7]

In June 2003 and June 2005 all (30,947 and 58,996) non-HPV-vaccinated, 18 and 18-19 year-old Finnish females from the entire, immediately adjacent birth cohorts, born respectively in Q3/1984-Q2/1985 and Q3/1985-Q2/1987, not eligible to the above-mentioned clinical trials, were invited to participate in Finnish Cancer Registry (FCR) –based follow-up (figure 1).[6,7] Receiving HPV-vaccination was an exclusion criterion. Respectively in June 2003 and June 2005 6,499 and 9,166 18-19 year-old non-HPV vaccinated females were enrolled (figure 1).[6,7] During Q4/2002-Q1/2003 additional 861 16-17 year-old placebo recipients of the FUTURE II trial[7] were enrolled (figure 1).

All the clinical trial participants and the non-HPV-vaccinated controls consented to the FCR-based follow-up for invasive, histopathologically confirmed cancer outcome.[1,6-10] The follow-up was based on unique Finnish personal identifiers available at our study database following the informed consent, and generally used at the FCR. It was age-aligned[8] to ensure that all the originally 16-17 years-old HPV-vaccinated women and originally 16-19 years-old non-HPV vaccinated women were of similar age during comparable follow-up time-period of 11 years between 2007 and 2019.

For peer review only

All participants of the FCR-based follow-up were invited to cervical screening at the ages of 25, 30 and 35 years. For those with abnormal cytology during the trials, there was referral to colposcopy with biopsy within 6 months[1] and the FCR follow-up thus started 6 months after the trials' end. All the cohorts responded at the age of 22 years to a life-style questionnaire, with special emphasis on sexual health and previous HPV-vaccination. Death and emigration were elimination criteria. For the non-HPV-vaccinated any identified HPV- vaccination was an elimination criteria. All the clinical HPV-vaccination trials, and the long-term follow-up of the trial cohorts and the non-HPV-vaccinated cohort were approved by the Finnish National Ethical Review Board (ERB, TUKIJA 1150/2002, 1153/2003, 1174/2004).

Patient and Public Involvement Patients were not involved in this preventive intervention study that involved originally adolescent HPV vaccinated or unvaccinated young women. To guarantee public involvement the entire target population was duly informed by letters using the up to date address service of the Finnish Population Register. The trial invitations were mailed to parents or guardians of all adolescents girls of the above-mentioned birth cohorts and living in the same house-hold. This was done even if the girl's own informed consent was sufficient for participation according to the Finnish law. Public involvement in the enrolment was further assured by information lectures given by the study nurses at parental evenings of the secondary high-schools and technical schools in the seven and eighteen trial communities.

Laboratory analyses The diagnostic histopathological blocks were requested from the pathology laboratories that had notified the FCR about the incident cancer cases according to a specific permission from the Finnish National Supervisory Authority for Welfare and Health (Valvira) without informing the patients. Before HPV DNA-typing the presence of neoplastic tissue was re-reviewed by two experienced pathologists. Blocks were sectioned according to a contamination-

For peer review only - http://bmjopen.bmj.com/site/about/guidelines.xhtml

proof manner. Extraction, amplification, and typing of the intralesional HPV DNA was performed as previously described.[11,12] HPV types that remained unknown were identified by sequencing.

Statistical analyses The enrolled HPV vaccinated and unvaccinated adolescents provided 80% power for the identification of vaccine efficacy (VE) against cervical cancer.[6,7] The VE was calculated on the intention-to-treat principle including all individuals (regardless of baseline HPV status) who had received at least one vaccine dose in the HPV-vaccine arm. Statistical software SAS 9.4 software (SAS Institute, Cary, NC) was used. The 95% confidence intervals (95% CI) were based on exact binomial distribution of number of vaccinated cases conditional on total number of cases.[13]

For peer review only

RESULTS

The age-aligned Finnish Cancer Registry follow-up for incident invasive cancers lasted by cohort for up to 11 years between November 2007 and Dec 2019. According to Finnish Population Census Register altogether 10 FUTURE II, 19 PATRICIA and 73 non-HPV vaccinated participants died during the follow-up (figure 1). It resulted in respectively 33,792 and 174,340 follow-up years for the HPV-vaccine and non-HPV vaccine cohorts.

No HPV-associated cancer cases were found in the 3,341 HPV-vaccinated females through the 33,792 women years of the follow up (table 1). The follow-up identified in the 16,526 non-HPV-vaccinated women 15 cases with invasive cervical cancer (ICC) in the 16,526 non-HPV vaccinated females during 174,320 women years of follow-up. Four of the ICC cases were adenocarcinomas and 11 were squamous cell carcinomas (SCC). One cervical SCC case, diagnosed late in 2019, was outside the age-aligned time-window of 11 years that guaranteed equal follow-up for the different cohorts. This case was not included in further analyses. The incidence of the 14 eligible ICC cases in the non-HPV-vaccinated women was 8.0 per 100,000 women years (table 1).

The single, invasive vaginal, vulvar and tongue cancer cases were all squamous cell carcinomas. Overall, in the non-HPV-vaccinated women the incidence of the HPV-associated cancers was 9.8 per 100,000 women years. Incidence rates of breast cancer, thyroid cancer and melanoma were essentially similar in all cohorts with highly overlapping 95% confidence intervals (table 1).

HPV DNA was found in all the identified and eligible 17 invasive HPV-associated cancer cases (table 2). HPV16 was present in 9 invasive cervical cancer cases. HPV16 was present in one female with vaginal cancer, who also had HPV16 positive cervical cancer. HPV18, HPV33 and HPV52 were each present in two ICC cases, and HPV45 in one ICC case. HPV66 (a vulvar cancer also positive for HPV52) and HPV213 (a tongue cancer) were present in one case each (table 2). With

For peer review only - http://bmjopen.bmj.com/site/about/guidelines.xhtml

no identified HPV-associated cancer cases in the HPV-vaccinated females, we found a vaccine efficacy of 100% (95% confidence interval 2,100; $p<0.05$) against all HPV DNA-positive invasive cancers.

For peer review only

For peer review only - http://bmjopen.bmj.com/site/about/guidelines.xhtml

DISCUSSION

We report the first randomized trial –derived evidence on the significant, 100% HPV-vaccine efficacy against HPV DNA-positive invasive cancers. We followed both the bivalent and the quadrivalent HPV-vaccine recipients for 11 years after the clinical trials had ended (up to 17 years post vaccination), and similarly-aged non-HPV vaccinated females, respectively over approximately 35,000 and 175,000 follow-up years by the population-based, quality-controlled Finnish Cancer Registry.[9,10] Vaccine efficacy estimates against invasive HPV16/18 positive cancers, and against invasive cancer cases positive for the bivalent vaccine cross-protected HPV types 33/45/52[14-16] were all 100%, albeit with very wide confidence intervals.

The significant 100% VE against all HPV-positive cancers is reassuring for both the bivalent and quadrivalent HPV-vaccines, three shots of which were given to, respectively 26% and 74% of the vaccinees in 2002 and 2004. A recent Swedish study[4] which was also based on accurate, high quality cancer registry follow-up[13] suggested that up to 12% of invasive cervical cancer cases may not be prevented by the quadrivalent vaccine. We have recently shown that long-term cross-neutralizing antibody responses induced by these two vaccines differ significantly.[16] Thus, continuation of the cancer registry-based long-term follow-up of the population-based bivalent and the quadrivalent HPV-vaccine cohorts versus non-HPV vaccinated cohorts[6,7] is warranted to understand if there were a possible difference their efficacy.

Population-based, age-aligned follow-up of trial cohorts and accurate, histologically confirmed, invasive cancer case definitions of the quality-controlled Finnish Cancer Registry[6-8,12,13,17] were pivotal strengths of this study. We could ensure identical cervical screening history of HPV vaccinated and non-HPV vaccinated cohorts for the 1983-1988 birth cohorts that were included in this study. The 5-year interval screening at ages 25, 30, 35… is according to local standard of care.

For peer review only - http://bmjopen.bmj.com/site/about/guidelines.xhtml

During 2007-2009 entire 1992-1995 birth cohorts of 33 Finnish towns were enrolled in our population-based community-randomized HPV-vaccination trial.[18,19] HPV16/18 vaccine recipients of that trial have since 2014 participated in a re-randomized screening trial at ages 22, 25 and 28 years[20], while their non-HPV vaccinated counterparts have not had this opportunity. These sizeable cohorts, >40,000 individuals in total, were not included in this study due to their gradually more and more different screening histories.

Two independent pathologists ensured that neoplastic tissue was present in the sections of the initial diagnostic biopsy blocks that we wanted to use for HPV testing. In three ICC cases, CIN3 was left in the diagnostic block that was used for HPV DNA typing but the sectioned material was deemed to adequately represent oncogenic HPV types involved. The blocks were found to be positive for HPV16, 33 and 16/52 DNA. Conceivably, due to the lesion size in the diagnostic block prior sections had removed an invasive cervical cancer before the new sections were made for the HPV typing.

Recruiting and enrolling the different cohorts in a population-based fashion during 2002-2005 was an important prerequisite for the country-wide cancer registry –based follow-up.[6-8,17] Due to the follow-up evidence on protection against the most stringent, invasive HPV-associated cancer end-points is now emerging more rapidly[4,5] than the evidence on the efficacy of HBV vaccination against hepatocellular cancer. [21] HPV-cancers in young adults may be more rapidly developing but establishing the trial cohorts and concomitant control cohorts, and their follow-up 17 years ago with identical possibilities for organized cervical screening at ages 25, 30 and 35 years[1,17] has been a strength of this study. At the time of HPV-vaccine licensure (2006/2007) the control cohort was above 20 years of age, and opportunistic vaccination was negligible but could not be totally ruled out[22,23] which should be listed as a limitation of this study. On the other hand, cross-vaccinating 50% of the FUTURE II placebo arm after age 20 no material effect on their CIN3 incidence.[8]

For peer review only - http://bmjopen.bmj.com/site/about/guidelines.xhtml

The excellent vaccine efficacy against HPV-positive cancers, now documented from a randomized study setting, is an important evidence of the long-term impact of HPV vaccination. It indicates that prevention of a sexually transmitted infection and associated cancer by prophylactic vaccination are doable, and paves the way for the WHO's initiative on the elimination of cervical cancer.[24]

For peer review only

Acknowledgements The review of diagnostic histopathological blocks for HPV DNA analysis by drs. Mensur Dzabic and Ralf Butzow is gratefully acknowledged.

Funding GSK Biologicals SA (HPV-027, 115006) and Finnish Cancer Foundation (MS790) supported the study financially,

Conflicts of interes We declare that ML, DA, JD and JP have received grants from Merck & Co. Inc. and/or from the GSK group of companies through their respective employers. GSK Biologicals SA was provided the opportunity to review this manuscript but the authors are solely responsible for final content and interpretation.

Contributorship The corresponding author attests that all listed authors meet authorship criteria and that no others meeting the criteria have been omitted. Matti Lehtinen, Tapio Luostarinen, Dan Apter, Joakim Dillner, Tiina Eriksson, Jorma Paavonen, and Eero Pukkala were involved in the conception and/or the design of the study. Matti Lehtinen, Dan Apter, Anne Bly, Tiina Eriksson, Katja Harjula, Kaisa Heikkilä, Mari Hokkanen, Heidi Karttunen, Marjo Kuortti, Tapio Luostarinen, Pekka Nieminen, Mervi Nummela, Johanna Palmroth, Tiina Petäjä, Anna Söderlund-Strand, and Ulla Veivo participated in the collection and generation of the study data. Tiina Eriksson, Penelope Gray, Camilla Lagheden and Tapio Luostarinen performed the analyses. Matti Lehtinen, Dan Apter, Tapio Luostarinen, Jorma Paavonen, and Eero Pukkala were involved in the analyses and/or interpretation of the data. All authors contributed to development of this manuscript, had full access to the data and gave final approval before submission.

Transparency declaration The leading author affirms that the manuscript is an honest, accurate, and transparent account of the study being reported; that no important aspects of the study have been omitted; and that any discrepancies from the study as planned and registered have been explained.

Data availability

Data is available upon request from corresponding author.

For peer review only - http://bmjopen.bmj.com/site/about/guidelines.xhtml

REFERENCES

1. Lehtinen M, Lagheden C, Luostarinen T, et al. Ten-year follow-up of human papillomavirus vaccine efficacy against the most stringent cervical neoplasia end-point – registry-based follow-up of three cohorts from randomized trials. *BMJ Open* 2017;7:e015867.

2. Kjaer SK, Nygard M, Sundstrom K, et al. Final analysis of a 14-year long-term follow-up study of the effectiveness and immunogenicity of the quadrivalent human papilloma virus vaccine in women from four Nordic countries. *EClinMed* 2020; 23:100401.

3. Kjaer SK, Nygard M, Sundstrom K, et al. Long-term effectiveness of the ninevalent human papilloma virus vaccine in Scandinavian women. *Human Vac. Immunother.* 2020; doi:10.1082/216455.2020.1839292

4. Lei J, Ploner A, Elfström KM, et al. HPV vaccination and the risk of invasive cervical cancer. *N Engl J Med* 2020;383:1340-8.

5. Luostarinen T, Apter D, Diller J, et al. Vaccination protects against invasive HPV-associated cancer. *Int J Cancer* 2018;142:2186-7.

6. Lehtinen M, Idänpään-Heikkilä I, Lunnas T, et al. Population-based enrolment of adolescents in a long-term follow-up trial of HPV vaccine efficacy. *Int J STD AIDS* 2006;17:237-46.

7. Lehtinen M, Apter D, Dubin G, et al. Enrolment of 22,000 adolescent women to cancer registry follow-up for long-term human papillomavirus vaccine efficacy. *Int J STD AIDS* 2006;17:517-21.

8. Rana M, Huhtala H, Apter D, et al. Understanding long-term protection of HPV vaccination against cervical carcinoma: Cancer registry-based follow-up. *Int J Cancer* 2012;132:2833-8.

9. Korhonen P, Malila N, Pukkala E, et al. The Finnish Cancer Registry as follow-up source of a large trial cohort-accuracy and delay. *Acta Oncol* 2002;41:381-8.

10. Pukkala E , Engholm G, Højsgaard Schmidt LK, et al. Nordic Cancer Registries - an overview of their procedures and data comparability. *Acta Oncol* 2018;57;440-55.

For peer review only - http://bmjopen.bmj.com/site/about/guidelines.xhtml

11. Lagheden C, Eklund C, Kleppe SN, et al. Validation of a standardized extraction method for formalin-fixed paraffin-embedded tissue samples. *J Clin Virol* 2016;80:36-9.

12. Lagheden C, Eklund C, Lamin H, et al. Nationwide comprehensive human papillomavirus (HPV) genotyping of invasive cervical cancer. Br J Cancer 2018; **118**: 1377-81.

13. Ewell M. Comparing methods for calculating confidence intervals for vaccine efficacy. *Stat Med* 1996;15:2379-92.

14. Wheeler CM, Castellsague X, Garland S, et al. Efficacy of the HPV-16/18 AS04-adjuvanted vaccine against cervical infection and precancer caused by non-vaccine oncogenic HPV types (PATRICIA trial). *Lancet Oncol* 2012;13:100-10.

15. Bogaards JA, vad der Weele P, Woestenberg PJ, et al. Bivalent HPV vaccine effectiveness correlates with phylogenetic distance from HPV vaccine types 16 and 18. *J Infect Dis* 2019;220: 1141-6.

16. Mariz F, Bender N, Eriksson T, et al. Sustainability of bi- and quadrivalent HPV vaccine-induced neutralizing antibodies. *Lancet Infect Dis* 2020; in press.

17. Lehtinen M, Herrero R, Mayaud P, et al. Studies to assess long-term efficacy and effectiveness of HPV vaccination in developed and developing countries. *Vaccine* 2006;24S:233-41.

18. Lehtinen M, Baussano I, Apter D, et al. Characteristics of a cluster-randomized, phase IV human papillomavirus vaccination effectiveness trial. *Vaccine* 2015;33:1284-90.

19. Vänskä S, Luostarinen T, Baussano I, et al. Vaccination with moderate coverage eradicates oncogenic papillomaviruses if a gender-neutral strategy is applied. *J Infect Dis* 2020;222:948-56.

20. Louvanto K, Eriksson M, Elfströn M, et al. Effectiveness of screening in human papillomavirus vaccinated women. *Int J Cancer* 2020;147:440-7.

21. McMahon BJ, Bulkow LR, Singleton RJ, et al. Elimination of hepatocellular carcinoma and acute hepatitis B in children 25 years after a hepatitis B newborn and catch-up immunization program. *Hepatology* 2011;54:801-7.

For peer review only - http://bmjopen.bmj.com/site/about/guidelines.xhtml

22. Woodhall SC, Eriksson T, Nykanen A-M, et al. Impact of HPV vaccination on quality of life. *Eur J Contracept Reproduct Health Care* 2011;16:3-8.

23. Eriksson T, Torvinen, Lehtinen MM, et al. Impact of HPV16/18 vaccination on quality of life. *Eur J Contracept Reproduct Health Care* 2013;18:364-71.

24. Basu P, Hutubessy R, Broutet N. Cervical cancer: WHO called for elimination, not eradication *BMJ* 2019;366doi:https://doi.org/10.1136/bmj.l5668.

For peer review only

Figure 1. Study flow chart defines the non-overlapping birth cohorts enrolled to long-term follow-up of human papillomavirus vaccination trials involving the quadrivalent HPV6/11/16/18 vaccine (FUTURE II) or the bivalent HPV16/18 vaccine (PATRICIA and HPV-012), or two non-HPV vaccinated control cohorts by population-based invitations via Finnish Population Register in 2002-05. Q=annual quartal.

For peer review only

For peer review only - http://bmjopen.bmj.com/site/about/guidelines.xhtml

Table 1. Numbers (n) and incidence rates (/100,000 person years) of human papillomavirus (HPV) associated invasive cancers and other common cancers in cluster-randomized cohorts of altogether 3,341 16-17 year-old female HPV16/18 or HPV6/11/16/18 vaccine recipients and 16,526 non-HPV vaccinated, originally 16-19 year-old females followed up§ between 2007 to 2019.

	HPV vaccinated women (33,792 person years)		Non-HPV vaccinated women (174,340 person years)	
End-point	n	Rate (95%CI)	n	Rate (95% CI)
Cervical cancer	0	-	14	8.0 (4.8, 13.6)
All HPV-positive cancers*	0	-	17	9.8 (6.1, 15.7)
Breast cancer	3	8.9 (2.9, 28)	27	15.5 (10.6, 23)
Thyroid cancer	2	5.9 (1.5, 24)	16	9.2 (5.6, 15.0)
Melanoma	8	23.8 (11.8, 47)	22	12.6 (8.3, 19.2)

§For corresponding age-aligned sub-cohorts, up to 11-years of passive follow-up was by the population-based Finnish Cancer Registry.
*14 cervical cancers, one vaginal cancer, one vulvar cancer, one tongue cancer

For peer review only - http://bmjopen.bmj.com/site/about/guidelines.xhtml

Table 2. Numbers (n) and incidence rates (/100,000 person years) of human papillomavirus (HPV) positive invasive cancers by HPV type in cluster-randomized cohorts of altogether 3,341 16-17 year-old female HPV16/18 or HPV6/11/16/18 vaccine recipients and 16,526 non-HPV vaccinated, originally 16-19 year-old females followed up§ during 2007 to 2019.

	HPV vaccinated women (33,792 person years)		Non-HPV vaccinated women (174,340 person years)	
End-point	n	Rate (95%CI)	n	Rate (95% CI)
Cervical cancer				
HPV16	0	-	9	5.2 (2.7, 9.9)
HPV18	0	-	2	1.1 (0.3, 4.6)
HPV16/18	0	-	11	6.3 (3.5, 11.4)
HPV33	0	-	2	1.1 (0.3, 4.6)
HPV45	0	-	1	0.6 (0.1, 4.1)
HPV52*	0	-	1*	0.6 (0.1, 4.1)
Any HPV	0	-	14	8.0 (4.8, 13.6)
Vaginal cancer				
HPV16	0	-	1	0.6 (0.1, 4.1)
Vulvar cancer				
HPV52#	0	-	1	0.6 (0.1, 4.1)
Tongue cancer				
HPV213	0	-	1	0.6 (0.1, 4.1)

§For corresponding age-aligned sub-cohorts, up to 11-years of passive follow-up was by the population-based Finnish Cancer Registry.
*Positive for both HPV16 and HPV52, #Positive for both HPV52 and HPV66

For peer review only - http://bmjopen.bmj.com/site/about/guidelines.xhtml

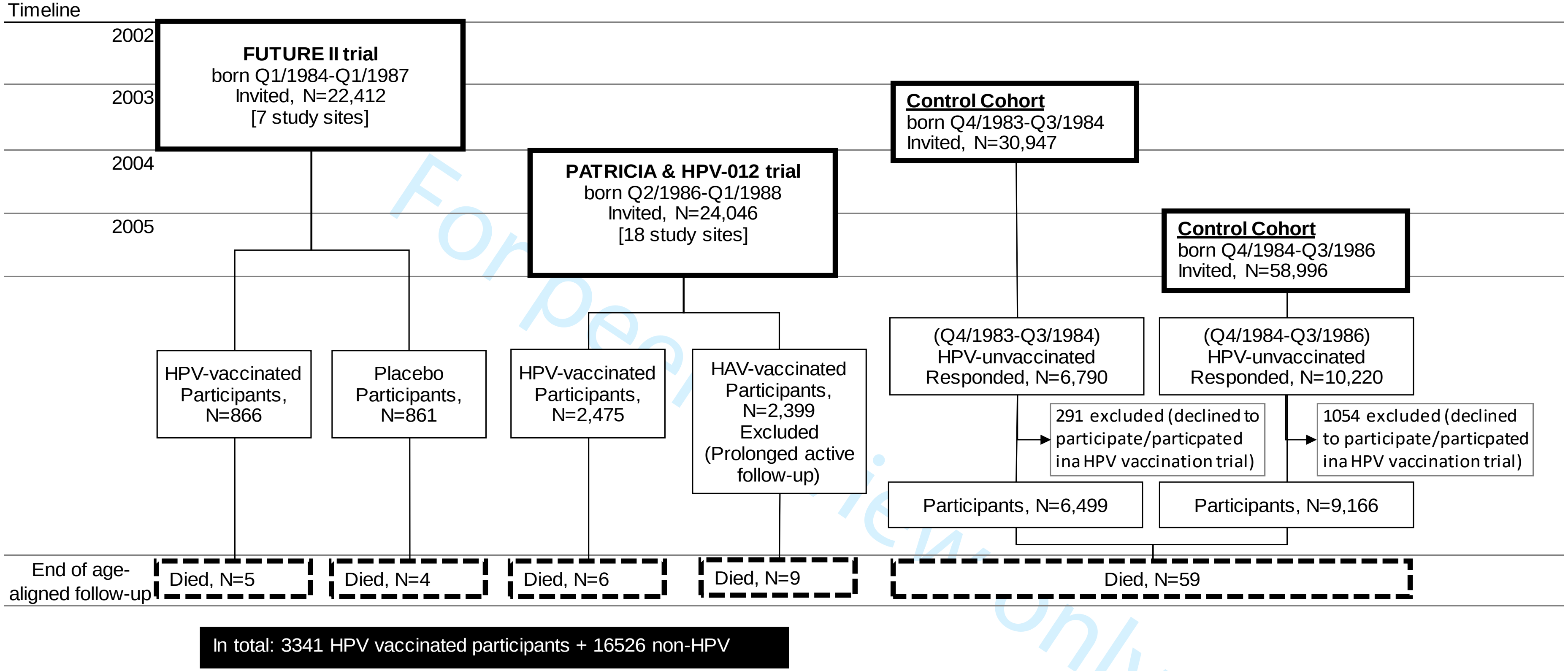
Timeline
2002
2003
2004
2005
FUTURE II trial
born Q1/1984-Q1/1987
Invited, N=22,412
[7 study sites]
PATRICIA & HPV-012 trial
born Q2/1986-Q1/1988
Invited, N=24,046
[18 study sites]
Control Cohort
born Q4/1983-Q3/1984
Invited, N=30,947
Control Cohort
born Q4/1984-Q3/1986
Invited, N=58,996
HPV-vaccinated Participants, N=866
Placebo Participants, N=861
HPV-vaccinated Participants, N=2,475
HAV-vaccinated Participants, N=2,399 Excluded (Prolonged active follow-up)
(Q4/1983-Q3/1984) HPV-unvaccinated Responded, N=6,790
(Q4/1984-Q3/1986) HPV-unvaccinated Responded, N=10,220
291 excluded (declined to participate/particpated ina HPV vaccination trial)
1054 excluded (declined to participate/particpated ina HPV vaccination trial)
Participants, N=6,499
Participants, N=9,166
End of age-aligned follow-up
Died, N=5
Died, N=4
Died, N=6
Died, N=9
Died, N=59
In total: 3341 HPV vaccinated participants + 16526 non-HPV

STROBE Statement—Checklist of items that should be included in reports of ***cohort studies***

	Item No	Recommendation	Page No
**Title and abstract**	1	(*a*) Indicate the study's design with a commonly used term in the title or the abstract	2
		(*b*) Provide in the abstract an informative and balanced summary of what was done and what was found	2
**Introduction**			
Background/rationale	2	Explain the scientific background and rationale for the investigation being reported	3
Objectives	3	State specific objectives, including any prespecified hypotheses	3
**Methods**			
Study design	4	Present key elements of study design early in the paper	3
Setting	5	Describe the setting, locations, and relevant dates, including periods of recruitment, exposure, follow-up, and data collection	4,5
Participants	6	(*a*) Give the eligibility criteria, and the sources and methods of selection of participants. Describe methods of follow-up	4,5
		(*b*)For matched studies, give matching criteria and number of exposed and unexposed	-
Variables	7	Clearly define all outcomes, exposures, predictors, potential confounders, and effect modifiers. Give diagnostic criteria, if applicable	4
Data sources/ measurement	8*	For each variable of interest, give sources of data and details of methods of assessment (measurement). Describe comparability of assessment methods if there is more than one group	4,5
Bias	9	Describe any efforts to address potential sources of bias	8,9
Study size	10	Explain how the study size was arrived at	5
Quantitative variables	11	Explain how quantitative variables were handled in the analyses. If applicable, describe which groupings were chosen and why	
Statistical methods	12	(*a*) Describe all statistical methods, including those used to control for confounding	5
		(*b*) Describe any methods used to examine subgroups and interactions	
		(*c*) Explain how missing data were addressed	
		(*d*) If applicable, explain how loss to follow-up was addressed	
		(*e*) Describe any sensitivity analyses	
**Results**			
Participants	13*	(a) Report numbers of individuals at each stage of study—eg numbers potentially eligible, examined for eligibility, confirmed eligible, included in the study, completing follow-up, and analysed	6
		(b) Give reasons for non-participation at each stage	6
		(c) Consider use of a flow diagram	6
Descriptive data	14*	(a) Give characteristics of study participants (eg demographic, clinical, social) and information on exposures and potential confounders	6
		(b) Indicate number of participants with missing data for each variable of interest	-
		(c) Summarise follow-up time (eg, average and total amount)	6
Outcome data	15*	Report numbers of outcome events or summary measures over time	6

For peer review only - http://bmjopen.bmj.com/site/about/guidelines.xhtml

Main results	16	(*a*) Give unadjusted estimates and, if applicable, confounder-adjusted estimates and their precision (eg, 95% confidence interval). Make clear which confounders were adjusted for and why they were included<br>(*b*) Report category boundaries when continuous variables were categorized<br>(*c*) If relevant, consider translating estimates of relative risk into absolute risk for a meaningful time period	6,7
Other analyses	17	Report other analyses done—eg analyses of subgroups and interactions, and sensitivity analyses	-
**Discussion**			
Key results	18	Summarise key results with reference to study objectives	8
Limitations	19	Discuss limitations of the study, taking into account sources of potential bias or imprecision. Discuss both direction and magnitude of any potential bias	8, 9
Interpretation	20	Give a cautious overall interpretation of results considering objectives, limitations, multiplicity of analyses, results from similar studies, and other relevant evidence	2, 9
Generalisability	21	Discuss the generalisability (external validity) of the study results	9
**Other information**			
Funding	22	Give the source of funding and the role of the funders for the present study and, if applicable, for the original study on which the present article is based	1

*Give information separately for exposed and unexposed groups.

Note: An Explanation and Elaboration article discusses each checklist item and gives methodological background and published examples of transparent reporting. The STROBE checklist is best used in conjunction with this article (freely available on the Web sites of PLoS Medicine at http://www.plosmedicine.org/, Annals of Internal Medicine at http://www.annals.org/, and Epidemiology at http://www.epidem.com/). Information on the STROBE Initiative is available at http://www.strobe-statement.org.

For peer review only

For peer review only - http://bmjopen.bmj.com/site/about/guidelines.xhtml
